# Gating by Functionally Indivisible Cerebellar Circuits: a Hypothesis

**DOI:** 10.1007/s12311-020-01223-6

**Published:** 2021-01-19

**Authors:** Mike Gilbert, Chris Miall

**Affiliations:** School of Psychology, University of Birmingham, Birmingham, UK

**Keywords:** Theory, Cerebellum, Learning, Memory, Purkinje cells, Parallel fibres

## Abstract

The attempt to understand the cerebellum has been dominated for years by supervised learning models. The central idea is that a learning algorithm modifies transmission strength at repeatedly co-active synapses, creating memories stored as finely calibrated synaptic weights. As a result, Purkinje cells, usually the de facto output cells of these models, acquire a modified response to input in a remembered pattern. This paper proposes an alternative model of pattern memory in which the function of a match is permissive, allowing but not driving output, and accordingly controlling the timing of output but not the rate of firing by Purkinje cells. Learning does not result in graded synaptic weights. There is no supervised learning algorithm or memory of individual patterns, which, like graded weights, are unnecessary to explain the evidence. Instead, patterns are classed as simply either known or not, at the level of input to a functional population of 100s of Purkinje cells (a microzone). The standard is strict. If only a handful of Purkinje cells receive a mismatch output of the whole circuit is blocked. Only if there is a full and accurate match are projection neurons in deep nuclei, which carry the output of most circuits, released from default inhibitory restraint. Purkinje cell firing at those times is a linear function of input rates. There is no effect of modification of synaptic transmission except to either allow or block output.

## Introduction

### What do we propose?

We argue that the cerebellum stores and consults memories at the level of functionally indivisible microzones - groups of hundreds of Purkinje cells which form part of its repeating internal circuits, whose output is received by a smaller group of nuclear cells which include the output cells of the circuit.

In the flocculus, the averaged firing rate of functionally grouped Purkinje cells (approximated by binning spikes across the population of recorded cells) has a linear, rapidly translated relationship with eye movement [43], with 3–5-ms temporal precision. Likewise, activity of groups of Purkinje cells in the oculomotor vermis precisely matches the metrics of saccades [24, 25].

Our scope is related but different. It is not correlation of Purkinje cell group codes with movement, at least directly, but how (and when) Purkinje cell firing is assured to be coordinated within a group/circuit, and the function of Purkinje cells outside those times. We argue that circuits are internally wired so that firing of a group must be tightly coordinated, or there is no response at all of the circuit as a whole. Pattern recognition effectively orchestrates functionally indivisible behaviour of the microzone, and thus the circuit. Input codes are contained in concurrent activity of large numbers of parallel fibres—that is, by simultaneous input to an entire population of Purkinje cells. Separate codes are used in pattern memory and control of output rates, expressed in group-coded properties that are independently variable. Contrary to 50 years of cerebellar theory, we argue that memory is collective, at microzone level, and that it gates output, but (otherwise) does not control Purkinje cell firing rates. These are instead controlled, in a time window opened by gating, by input rates.

### Background—the consensus

Purkinje cells, which exclusively carry the output of the cerebellar cortex, receive excitatory contact from parallel fibres and climbing fibres and inhibition from local interneurons. Parallel fibres are the axons of granule cells, which in turn receive contact from mossy fibres, which, with climbing fibres, provide the only glutamatergic input to the cerebellum. The architecture of this arrangement is thought to be repeated in all circuits. The dendritic field of Purkinje cells is very severely flattened in the plane perpendicular to the surface of the cerebellum and the long axis of folia. Parallel fibres lie parallel to the surface of the cerebellum and each other, and orthogonal to Purkinje cells, so that if the cerebellar cortex was unfolded and laid flat they would lie in (very numerous) parallel straight lines passing at right angles through dense ranks of Purkinje cells.

**Fig. 1:**
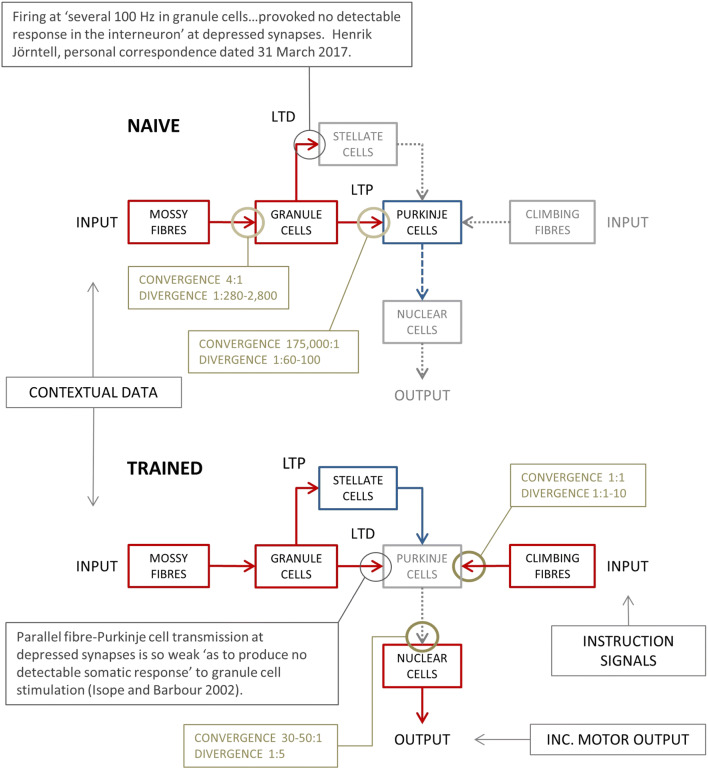
Schematic wiring diagram of the (much simplified) cerebellar circuit. Output of the circuit results only if mossy fibre input to the system recodes as internal signals traffic received at training-modified parallel fibre synapses on Purkinje cells and inhibitory interneurons. (Top) The default state. In this state, granule cell transmission to Purkinje cells is robust and to stellate cells is very weak. Purkinje cell firing at high spontaneous rates, elevated by excitatory input, inhibits nuclear cells—the output cells of the circuit. Red boxes and solid arrows: active glutamate neurons; blue and dashed arrows: active GABA neurons; grey and dotted arrows: silent neurons. (Bottom) Repeated pairing of convergent parallel fibre and climbing fibre input to a Purkinje cell teaches long-term modification of parallel fibre synaptic transmission. Learning accordingly modifies the response to a repeat of parallel fibre input in the same pattern of active cells. Training reverses the sign of learning at both synapse types. Partly as a result, firing of Purkinje cells is weakened or suspended in the conditioned response, causing a phasic reduction of inhibition by Purkinje cells of nuclear cells

Near coincidence of climbing fibre and parallel fibre stimulation (therefore ‘paired’) induces long-term depression of the parallel fibre-Purkinje cell synapse [21, 28, 48]; we term a synapse modified by this protocol ‘trained’. Learning models of the cerebellum propose that, following training, Purkinje cells acquire a learned response to input in a ‘known’ (i.e. repeatedly paired) pattern of active parallel fibres as a result of synaptic weight adjustments trained under climbing fibre tuition [1, 8, 14, 18]. Learning displaces the original, naïve response to input in a recognised configuration of active cells, which in that sense the system remembers. Pattern memory, stored as graduated synaptic weights, accordingly also provides control of Purkinje cells in the learned response. Weights are adjusted by a learning algorithm (but there is not a consensus which one). Figure 1 shows a (greatly) simplified circuit wiring diagram.

Models sharing these ideas, together with the idea that climbing fibre signals provide feedback about performance, have a long history [54]. Feedback typically takes the form of error signals [47] which signal the difference between actual and desired performance. Purkinje cells are thought to individually ‘most probably require specific error signals and learn heterogeneously’ [64], and are often treated as de facto output cells of the system.

### New proposals in more detail

This paper argues instead that:

I. The cerebellum stores memories at the level of functionally indivisible groups of hundreds of Purkinje cells, which form part of the cerebellar circuit. A group occupies a long thin strip of the cerebellar cortex (a microzone, 15–20 mm long and a few cells wide [41]).
II. The output of a group, received by the output cells of the circuit, is wired so that firing of the whole Purkinje cell population must be tightly coordinated, or there is no response at all of the circuit as a whole. Put another way, it effectively orchestrates functionally indivisible behaviour of the group, and thus the circuit.
III. Input to a circuit is coded at the collective levels of hundreds of signals received by each Purkinje cell and thousands received by the population of a microzone, at around the same time.
IV. Separate codes are used in pattern memory and control of output rates. The codes are contained in the same group activity of parallel fibres but in different collectively expressed parameters that are independently variable, permitting pattern recognition and control of Purkinje cell firing to proceed side by side, without mutual interference.
V. The response to a recognised pattern is permissive, allowing but not driving output. Circuit selection by pattern recognition controls timing of output and where it is sent, but not (otherwise) firing of Purkinje cells (or the output cells of the circuit)—in conflict with the supervised learning model, where learning codes output. To expand on this, we propose that training opens a time window where the ‘learned’ response of Purkinje cells—a phasic reduction of the simple spike rate—is a proportionate response to inhibition by interneurons controlled by granule cell rates (or a group code using rates), unmodified by parallel fibre synaptic weights. The function of weights, and learning, is to open the window and not (otherwise) to control the simple spike rate.
VI. An input pattern must be recognised by all of the Purkinje cells in a functional group to evoke a response of the circuit. There is no graded or intermediate response to a partial match (otherwise, a partial match would have an arbitrary outcome).
VII. Remembered patterns are not recognised individually—the response does not depend on a match between input in a particular pattern of active parallel fibres and a specific corresponding set of highly trained synapses. The cerebellum remembers learned patterns only as a class. The response discriminates between the class of known patterns as a whole and the (unlimited) residual class of all other patterns.
VIII. This does *not* confine the circuit to a binary response. Pattern memory and control of firing rates are separate and independently executed functions, though closely related. Control of Purkinje cell firing is by granule cell rates, indirectly but unfiltered, with learned timing provided by memory. Learning gates output, and rates are controlled independently, ad hoc.
IX. There is no supervised algorithm—for so long a mainstay of cerebellar modelling—which, like graded synaptic weights, is unnecessary to explain the evidence. The effect of learning is instead to polarise synaptic weights so that, at the scale of input to a Purkinje cell, they are functionally binary.
X. Without graded synaptic weights, there is no need for hypothetically heterogeneous, or graded, climbing fibre signals to teach them. Instead, functionally indivisible circuits receive functionally indivisible, binary, instruction signals. This is likely to be contentious. In our view, the evidence does not establish that graded lessons are coded in the number of spikes in a climbing fibre burst. In fact, it more naturally argues the reverse, that climbing fibre signals are not graded. We argue for a rehabilitation of the all-or-nothing climbing fibre signal (knowing this is an unpopular view).

## A small number of Purkinje cells is sufficient to strongly inhibit a whole nuclear group

### Anatomy

Substantially, all of the output of the cerebellar cortex which converges on a functional group of nuclear cells is from the same microzone or the same functional but dispersed group of microzones which form part of a multizonal circuit [2, 42]. Unless otherwise stated, ‘nuclear cell’ is used as shorthand for excitatory projection neurons (although deep nuclei also contain other cell types), and ‘circuit’ as a synonym of microcomplex, including a multizonal microcomplex (a circuit that contains more than one microzone) [2].

**Fig. 2:**
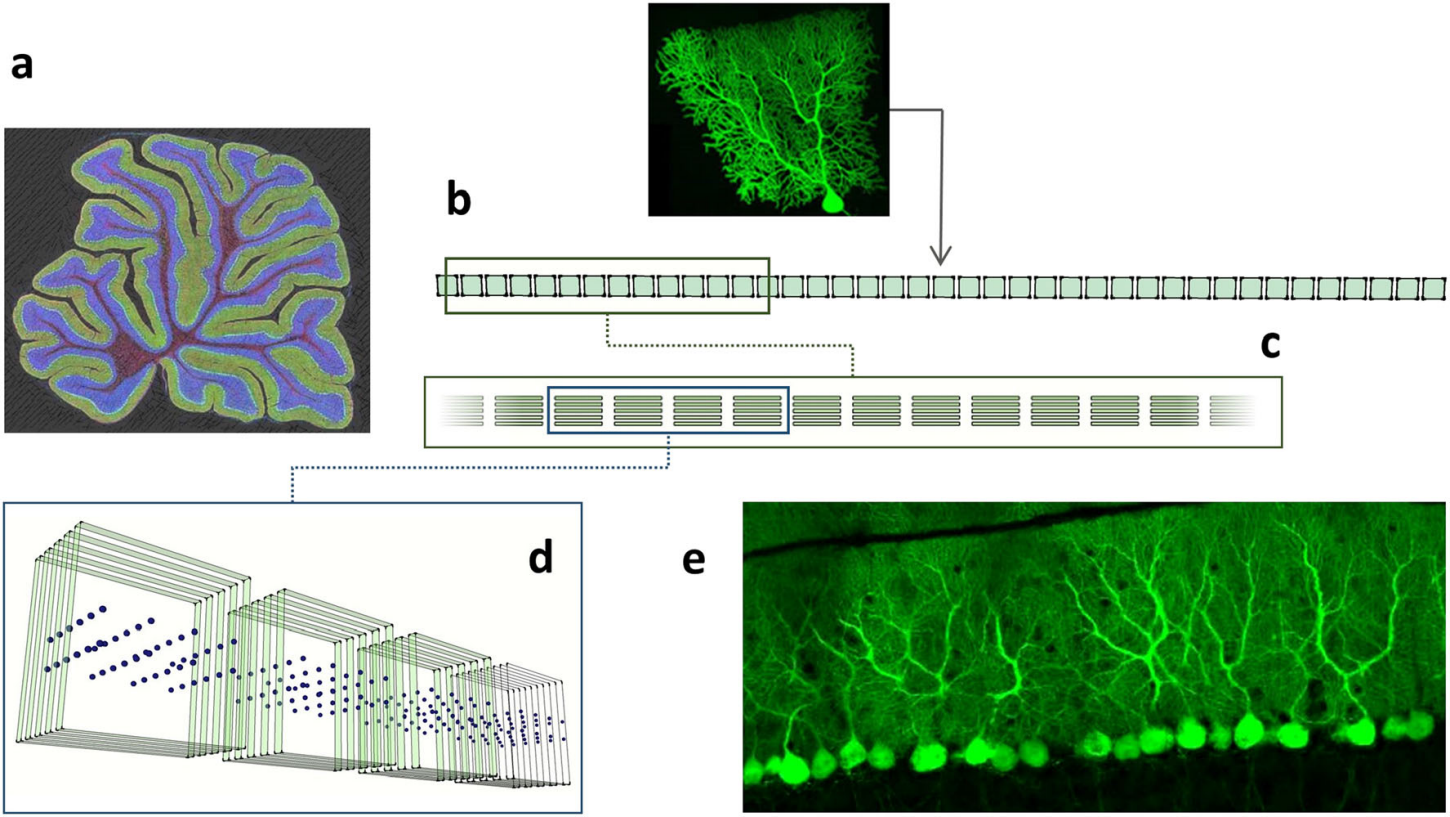
Graphic to illustrate the shape and dimensions of a microzone and a Purkinje cell. (a) Sagittal section of the rat cerebellum (obtained by multi-photon microscopy, courtesy Tom Deerinck) showing the very nearly always equal depth of the molecular layer. (b) Schematic of a sagittal row of 40 Purkinje cell dendritic territories, which form the molecular layer, the estimated length of a C3 microzone in the same plane as the slice in (a) but straightened out. (Purkinje cell image: Boris Barbour, with permission.) (c) An inset of (b) viewed from above. Microzones are just a few cells wide. Here, a row of 5 Purkinje cells spans a microzone from side to side. Although formalised, relative Purkinje cell and microzone dimensions are preserved. (d) Inset of (c), this time a perspective of 4 rows of Purkinje cells. The matrix of dots, representing stellate cell bodies, gives an idea of the number of stellate cells that occupy the space between Purkinje cells. Superficial stellate cells and basket cells are not shown. (e) The actual appearance of Purkinje cells in a sagittal slice (two-photon laser scanner micrograph, courtesy Mike Häusser)

Purkinje cells fire spontaneously at robust rates [10, 23, 49, 65]. They individually make powerful inhibitory contact on each of their nuclear targets via many boutons, each containing multiple synaptic densities [45, 59], averaging a total of perhaps 220–320 synaptic densities (24–36 boutons at an average of 9.2 ± 1.3 densities per bouton). Purkinje cells outnumber nuclear cells by around 10 to 1; each Purkinje cell makes contact on 4 or 5 nuclear cells, so that the convergence ratio is about 30–50:1 (rats: [45]) (Fig. 2).

The strong firing of Purkinje cells and their individually strong contact on nuclear cells mean that a single Purkinje cell may significantly impact on firing of its targets [44]. Modulation of nuclear cell firing requires the ‘substantial co-modulation of a large proportion of the PCs [Purkinje cells] that innervate the cell’ ([5], abstract). Put another way, less than that substantial proportion is insufficient for an effect on firing, so that a nuclear cell instead continues to receive an overriding influence of the small number whose firing is out of step. For example, the effect of a widespread and coordinated fall in rates may nonetheless be overridden by a minority which continue to fire at high intrinsic rates.

It is thought (but unconfirmed) that microcircuits may be the smallest functional division of the cerebellum. That is, the output of a microzone is not further divided into subgroups of Purkinje cells that make segregated contact on nuclear cells. ‘To date, there is no evidence to support [the idea] that different PCs [Purkinje cells] of the microzone control specific CN [cerebellar nuclei] cells within the micro-group [associated group of nuclear cells]’ (Bengtsson and Jorntell in [3] p. 663). If there is no internal organisation, a Purkinje cell makes contact at random on any 4 or 5 nuclear cells in the nuclear target group, and a nuclear cell receives contact from a random sample of 30–50 Purkinje cells.

### Quantifying random contact

Random contact can be quantified. Assuming divergence of 1:5, 5 Purkinje cells may inhibit, at high spontaneous frequency, as much as half a nuclear group of 50, making around 200–300 synapses on each nuclear cell—substantial inhibitory drive, from only 1.25% of the 400 Purkinje cells in the microzone (derived from cell and contact ratios). Adding 5 more active Purkinje cells would result in inhibition by 10 Purkinje cells of 5–50 nuclear neurons representing 10–100% of the target nuclear group, at 200–300 synapses per cell if input is distributed to 100% of the group, or up to 2,000–3,000 synapses in the also improbable event that input converges on just 10% of the nuclear group, and so on. These examples are intended to illustrate that a very small fraction (1.25–2.5%) of the afferent population of Purkinje cells may powerfully inhibit a majority of a nuclear group.

These are specific examples, but we can calculate the expected number of nuclear cells out of a group of 50 that receives contact from at least 1, 2 or 3 Purkinje cells out of a subset selected at random from the population with input to a nuclear group. The particular subset does not matter, assuming contact is at random. The size of the Purkinje cell population also does not affect the result. Variables that affect the result are the size of a subset and the divergence ratio.

For divergence of 1 to *d* and a nuclear group of *n*, the probability of contact on a particular nuclear cell by a single Purkinje cell is *d*/*n*. The probability that a particular nuclear cell receives contact from *x* out of *y* active Purkinje cells is given by $$ P(x)=\left(\begin{array}{c}y\\ {}x\end{array}\right)\times {\left(d/n\right)}^x\times {\left(1-\left(d/n\right)\right)}^{y-x} $$. The probability that a particular nuclear cell receives contact from *x* = 1 or more Purkinje cells is 1 − *P*(0), multiplied by *n* for the expected number of nuclear cells receiving it. The probability for *x* = 2 or more is 1 − *P*(0) − *P*(1), again multiplied by *n* for the expected number, and for *x* = 3 or more is 1 − *P*(0) − *P*(1) − *P*(2), and so on.

**Fig. 3:**
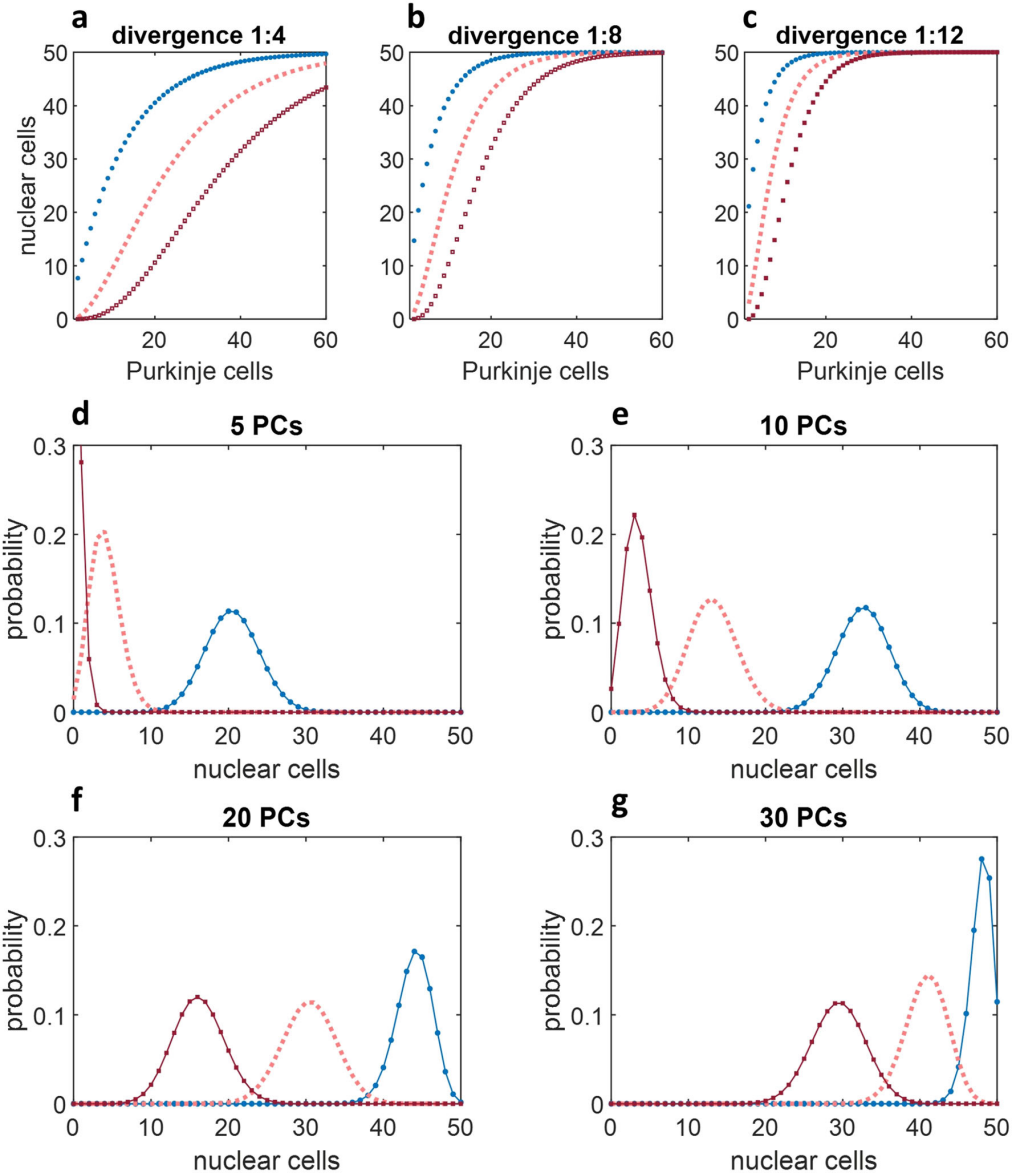
**a**–**c** The expected number (p*n) of nuclear cells, *y*, out of *n* = 50, that receive contact from 1 or more (blue data), 2 or more (pink) or 3 or more (red) out of *x* Purkinje cells, with divergence of 1:4 in **a**, 1:8 in **b** and 1:12 in **c**. A steeper curve indicates that fewer active Purkinje cells are needed to inhibit a nuclear group. Estimated average divergence for rats is 1:4–5, but Purkinje cells may influence (via nuclear interneurons, for example) a larger number of nuclear cells than only the ones they contact directly. **d**–**g** The probability, *y*, that *x* out of 50 nuclear cells receives contact from 1 or more (blue data), 2 or more (pink) or 3 or more (red) Purkinje cells, out of 5 in **d**, 10 in **e**, 20 in **f** and 30 in **g**, with divergence of 1:5. The significance is that a modest number of Purkinje cells is sufficient to statistically assure that almost all nuclear cells receive contact, and therefore to block output of the whole circuit (Purkinje cells make individually strong contact on nuclear cells). The number may be still lower if interneurons effectively increase divergence (shown in Fig. 4)

The results are shown in Fig. 3a–c. A steeper curve indicates that fewer active Purkinje cells are needed to inhibit a nuclear group. Efficiency is in proportion to the divergence of Purkinje cell contact on nuclear cells. The estimated divergence ratio in rats is 1 to 4 or 5. Higher ratios are shown because it will be proposed that in addition to a direct effect on nuclear cells Purkinje cells have an indirect effect via control of excitatory interneurons, so that it has an effect on a larger total than the number it contacts directly.

The expected number of inhibited nuclear cells is not a reliable prediction of the actual proportion on any particular occasion because the numbers are too low to express probability without a degree of random variation. Figure 3d–f show the distributed probability that any particular number of nuclear cells receive contact from a minimum or 1, 2 or 3 active Purkinje cells. Again, the effect depends on the number rather than the proportion of active Purkinje cells. There is a normal distribution for each condition, with a very reliably limited range, and the higher probabilities are in a narrower range centred on the middle of the distribution. The range becomes compressed at low and high numbers of nuclear cells.

If *d* = 5 and *n* = 50, the probability of contact on a particular nuclear cell by a particular Purkinje cell is $\frac{5}{50}=$ 0.1. The probability that a particular nuclear cell receives no contact from any of *y* Purkinje cells is therefore *P*(0) = (1 − 0.1)^*y*^, and contact from exactly one is $$ P(1)=\left(\begin{array}{c}y\\ {}1\end{array}\right)\times 0.1\times {\left(1-0.1\right)}^{y-1} $$, and the probability of contact from 1 or more, or *P*(≥1), is 1 − 0.9^*y*^. $$ P\left(\ge 2\right)=1-{0.9}^y-\left(\left(\begin{array}{c}y\\ {}1\end{array}\right)\times 0.1\times {0.9}^{y-1}\right) $$ and so on.

For contact from at least *x* out of *y* Purkinje cells on *z* out of *n* nuclear cells,

$$ \left(\begin{array}{c}n\\ {}\mathrm{z}\end{array}\right)\times {\left(1-P(0)-P(1)-\dots -P\left(x-1\right)\right)}^z\times {\left(P(0)+P(1)+\dots +P\left(x-1\right)\right)}^{n-z} $$

$$ \mathrm{where}\ P(x)=\left(\begin{array}{c}y\\ {}\mathrm{x}\end{array}\right)\times {p}^x\times {\left(1-p\right)}^{y-x} $$

where *p* =*d*/*n*.

For values of *y* greater than the number of Purkinje cells which converge on a nuclear cell, the calculation must be adjusted, because it becomes possible that input to some nuclear cells is saturated (i.e. all afferent Purkinje cells are active), affecting the probability of contact on others. Assuming convergence of 30–50:1, *y* must exceed at least 30 and perhaps as much as 50 to make an adjustment necessary. The amount of an adjustment would be very small at values not much higher than the convergence ratio. Nonetheless, partly for this reason, the range of the calculations is limited to 30.

To reformulate the calculation, the probability distribution of contact by multiple Purkinje cells onto nuclear cells is derived from the probability that a given nuclear cell (out of a group of *n* = 50) receives contact from *x* out of *y* Purkinje cells:

$$ P(x)={\left(\frac{d}{n}\right)}^x\times {\left(1-\frac{d}{n}\right)}^{y-x}\times \left(\begin{array}{c}y\\ {}x\end{array}\right) $$

with a Purkinje cell to nuclear cell divergence of 1 : *d*. Then the probability that a number of nuclear cells, *z*, receives contact from at least *x* out of *y* Purkinje cells is:

$$ P\left(x\  or\ more\right)={\left[1-\sum \limits_{j=0}^{x-1}P(x)\right]}^z\times {\left[\sum \limits_{j=0}^{x-1}P(x)\right]}^{n-z}\times \left(\begin{array}{c}n\\ {}z\end{array}\right) $$

The results are that a modest number of Purkinje cells is sufficient for a large majority of Purkinje cells to receive strong inhibition. This simple idea is unpacked in the next section.

### A Purkinje cell veto

The anatomy of contact by Purkinje cells on the output cells of the cerebellar circuit allows us to draw inferences about its function, assuming random distribution of contact by a Purkinje cell within a nuclear group.

1. Inhibition by Purkinje cells of nuclear cells is broadly equally distributed among a nuclear group even at low numbers of active Purkinje cells, so that the inhibition of a nuclear group reaches functional saturation efficiently. Accordingly, low Purkinje cell numbers are sufficient for inhibition at high spontaneous rates of a statistically assured high fraction of nuclear cells. These can be any Purkinje cells of the necessary minimum number.

By adding a proposal, we can infer (2) to (4) below. The proposal is that if firing of only a few Purkinje cells is out of step with the rest, so that they fire at unfettered rates, it overrides an effect of the others (so that there is a ‘veto’ of output of the circuit). This is not without support—it has been reported that the firing rate of most Purkinje cells which inhibit a nuclear cell must change in the same direction at the same time to cause an effect on the nuclear rate [5]—but has not been fully quantified. A veto is consistent with (and would explain) individually strong inhibition by Purkinje cells of nuclear cells, and a number of highly specialised synaptic adaptations discussed in the next section.

2) The coordinated suppression of Purkinje cell firing across the whole population of a microzone is necessary in order to disinhibit a nuclear cell group, because less than full coordination means that each nuclear cell is individually at high risk of receiving strong inhibition.
3) Therefore, at least in theory, a handful of Purkinje cells is sufficient to block the output of the whole circuit, because it is sufficient to override an effect on nuclear firing of the rest of the population.
4) Most of the inhibition of an individual nuclear cell is functionally supernumerary for most of the time. This is necessary in order that it can be any group of Purkinje cells which blocks output of the circuit if they do not participate in the conditioned pause [30, 52], regardless of the behaviour of the rest of the group.

## Adaptations facilitating a strong effect by a handful of Purkinje cells

This section discusses evidence that circuits are highly adapted to allow a few Purkinje cells to have a power of veto, and to mitigate unwanted side effects.

### Inhibitory bottleneck

Contact by Purkinje cells on nuclear cells ‘is characterised by preferential targeting of cell somata rather than dendrites’ ([61], p. 3443), while the majority (75%) of excitatory inputs are distal [13]. Purkinje cell synapses are therefore positioned to block an effect of excitatory input onto nuclear cells, exercising a powerful inhibitory veto.

### Nuclear interneurons

Deep nuclei contain excitatory (presumed glutamatergic) interneurons that fire spontaneously and which are inhibited by Purkinje cells [62]. Accordingly, they provide spontaneous drive to their targets, subject to Purkinje cell restraint. Assuming those targets include significant contact on the output cells of the circuit, Purkinje cells thus have an indirect as well as a direct modulatory influence on output (because they both directly inhibit nuclear cells and block tonic excitation). The effect of a reduction of a coordinated Purkinje cell rate is the reverse—output cells are released from inhibition and interneurons are released to excite them.

**Fig. 4:**
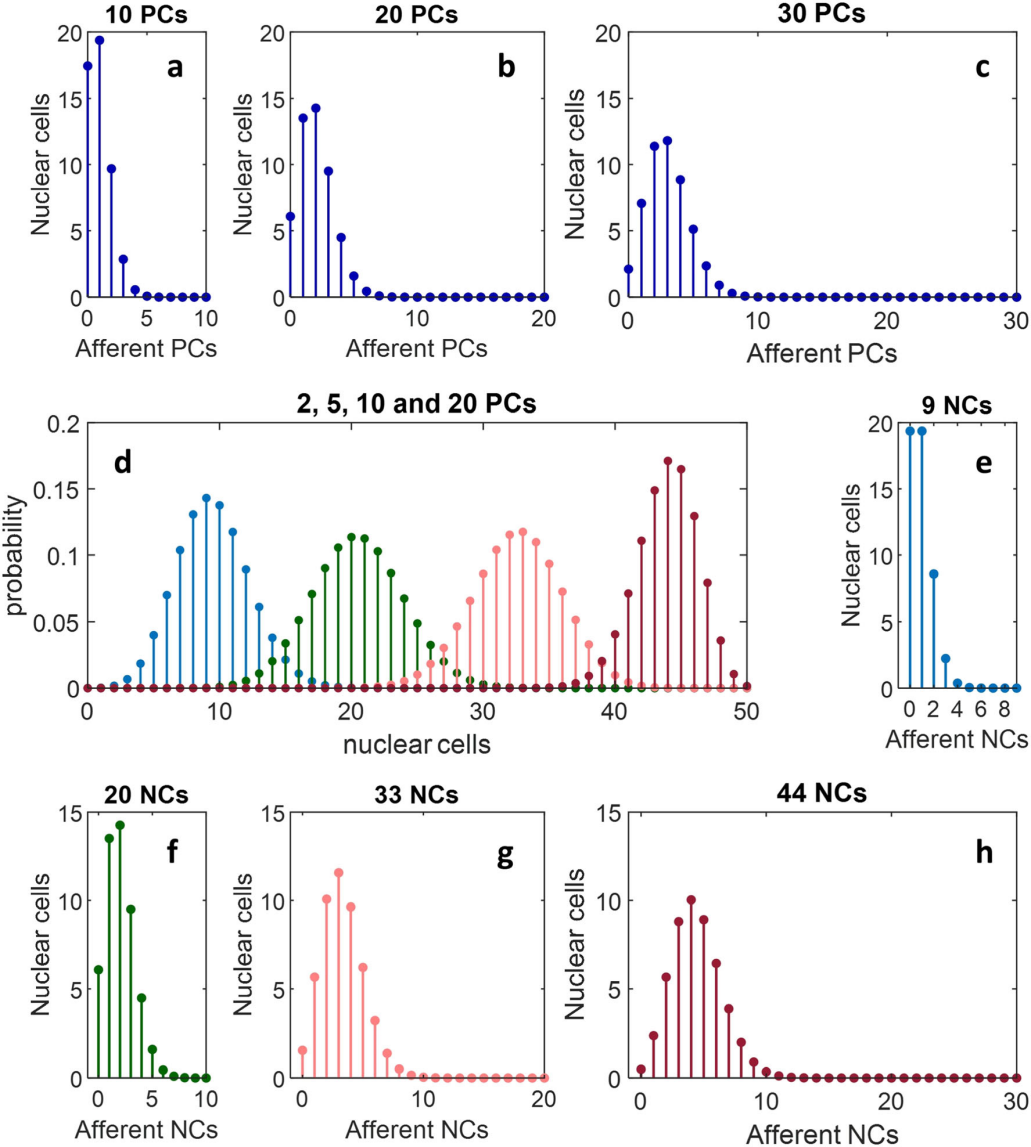
**a**–**c** The expected number of nuclear cells, *y* (out of *n* = 50), that receive contact from *x* out of a randomly selected subset of 10, 20 and 30 Purkinje cells, respectively, with divergence of 1:5. The number of Purkinje cells in these examples is a low fraction of the afferent population but still has good coverage of a nuclear group. For example, assuming an afferent population of 400 Purkinje cells (derived from divergence of 1:5 and convergence of 40:1), 2.5% (10, in **a**) make contact on an expected 64% of nuclear cells. Nonetheless, a very low number of Purkinje cells is likely to mean a significant fraction of nuclear cells receive no contact/effect. **d** The probability, *y*, that *x* out of *n* = 50 nuclear cells receive contact from at least 1 out of a randomly selected subset of 2 (blue), 5 (green), 10 (pink) and 20 (red) Purkinje cells (same divergence). This is a measure of coverage by divergence in one step, Purkinje cells onto nuclear cells, and also provides data used in panels **e**–**h**. **e**–**h** The expected number of nuclear cells, *y* (out of *n* = 50), that receive nucleo-nuclear collateral contact from *x* out of a randomly selected subset of 9, 20, 33 and 44 nuclear cells, the average (nearest whole) number receiving contact from at least 1 Purkinje cell in each of the conditions in panel **d**. Divergence—here of a nuclear cell onto neighbouring nuclear cells—is again 1:5 (assumed, because the ratio is unknown). The second step means that effective divergence of Purkinje cells onto nuclear cells is substantially higher than it is at each step. For example, just 2 Purkinje cells are sufficient for effective divergence onto an expected 60% of nuclear cells, 5 diverge onto 88% and 10 diverge onto an expected 96%. Moreover, these figures do not include nuclear cells they contact directly. Ten is sufficient for around 84% of a nuclear group to receive modulation of *convergent* collateral input, i.e. from two (or more) other nuclear cells, in addition to 48–82% that receive a direct effect

Interneurons may increase functional divergence of Purkinje cells onto nuclear projection neurons which carry the output of the circuit, because a single Purkinje cell both inhibits projection neurons on which it makes direct contact and also weakens or blocks tonic excitation of a second subset of those cells (which may overlap with the first). Assuming nuclear interneurons receive contact from a random sample of Purkinje cells and output cells receive contact from a random sample of interneurons, functional divergence of Purkinje cells onto output cells is amplified. The convergence and divergence ratios of Purkinje cells onto interneurons and of interneurons onto output cells are unknown (and the internal circuitry of deep nuclei generally is poorly understood). Accordingly, this idea awaits experimental corroboration. However, we can show that, if this pathway exists, there is a statistically assured effect on the size of the subset of nuclear cells that receive an influence of a Purkinje cell, and the effect is large at low divergence (Fig. 3a–c and Fig. 4).

The excitatory function of interneurons may be supplemented—or, conceivably, carried out^1^—by axon branches of nuclear projection neurons that terminate within deep nuclei, therefore with an interneuronal effect. If excitatory projection neurons make collateral contact on each other, the statistical effect should be similar to that achieved through interneurons. Figure 4e–h model the increase in de facto divergence mediated by nuclear collaterals. To model an interneuron pathway, we would need to assume the unknown ratio of excitatory interneurons to nuclear projection neurons and also the unknown divergence ratios of Purkinje cells onto interneurons and interneurons onto nuclear cells. By modelling collaterals, we instead only need assume the nucleo-nuclear divergence ratio.

The significance is that high divergence moves the Fig. 3d–g probability distributions to the right, where there is low uncertainty (narrow peak), and high participation of nuclear cells, and non-participation is confined to a low number. Accordingly, it assists an emphatic veto by a low number of Purkinje cells.

GABA/glycinergic nuclear interneurons [26], and possibly others [61], have also been reported. However, they probably receive light or no inhibition from Purkinje cells [61], so that there is no equivalent (but opposing) effect mediated by inhibitory interneurons.

### Adaptations of Purkinje cell-nuclear cell contact

Contact by Purkinje cells on nuclear cells is adapted to the barrage of inhibition received by nuclear cells and mitigates the heavy demands on Purkinje cells. Nuclear neurons fire spontaneously in vitro, with synaptic inputs removed or blocked, at an average of around 90 Hz (interpositus in mice: 70 Hz in males, 110 Hz in females, hence the average: [37, 46]). In vivo, under inhibition, they continue to fire but at substantially lower rates: 10–20 Hz in resting animals [58].

Contact is somatic and transmission is mediated by spillover confined to multisynaptic boutons. Boutons are adapted to provide a reliable and fast response, and mitigate depletion of the neurotransmitter supply, and (not reported but proposed) to prevent an effect leaking to other nuclear groups. Adaptation is in the form of ‘many specializations…[which include] large boutons, glial ensheathment, GABA transporters confined to astrocytes [at the bouton perimeter, and] multiple release sites’, and an enormous number of synaptic vesicles ([59] p. 123). The effect is that the nuclear response is bidirectionally sensitive at fast times with a high response probability of postsynaptic receptors.

On the face of it, strong inhibition by Purkinje cells might be expected to hold nuclear cells in an inactive state of hyperpolarisation. Transmission is weakened by short-term depression [44, 58–60]. A presynaptic form of depression may reduce neurotransmitter depletion [44].

It is an unreported but proposed effect of the same arrangement that it restricts diffusion of extrasynaptic GABA (because it is confined to boutons), thereby limiting an effect to the nuclear group that receives contact, and preventing an effect on other groups, even if group boundaries contain some intermingling. Otherwise, independent and functionally discrete control of nuclear groups would receive interference from diffusion of spillover from other groups.

## What is a veto for?

Why are circuits engineered so that a small number of Purkinje cells has a power of veto?

Input to the cerebellum terminating as mossy fibres on granule cells is thought to be recoded at the point of entry, in the granular layer, in the sense that it drives firing of a decorrelated permutation of active granule cells which is maintained homeostatically at a low and stable fraction of the large granule cell population, regardless of the number of mossy fibres driving it [6, 9], thus generating a random distribution and shifting pattern but low and stable level of parallel fibre activity. A single Purkinje cell may receive contact from 175,000 parallel fibres [39], so that even if a very low fraction of parallel fibres is active a single Purkinje cell receives several hundred inputs.

Microzones are defined by their climbing fibre input, so that all Purkinje cells in a microzone receive climbing fibre instruction as a volley, at the same time. Assuming a fixed density and random distribution of active parallel fibres, a climbing fibre volley is reliably paired with parallel fibre input received along the whole length of a microzone. Synaptic training with paired input accordingly teaches depression not at several hundreds of active synapses on a single Purkinje cell, but tens of thousands, on hundreds of Purkinje cells, which occupy a long thin strip that populates a microzone.

Following training, Purkinje cells respond to a known pattern with a phasic reduction of their firing rate and sometimes a full pause [30, 52]. Nuclear cells are sensitive to the dynamics of the simple spike rate [44, 58] and respond to a falling rate with an increase in their own firing. However, if a low number of Purkinje cells is enough for an override, non-participation of only a very modest region of a microzone—because it does not receive parallel fibre input in a known pattern—is sufficient for a veto. If part of a pattern of input to a microzone is known and part is not, there is a veto.

The result is the same regardless of the size of the region that receives the unknown part, provided it contains the necessary minimum number of Purkinje cells. As we might expect that Purkinje cells which are aligned on beam and therefore sample the same parallel fibre activity respond *en bloc*, the size (in the sagittal plane) of a region that contains the necessary number may be very modest—conceivably a single row of Purkinje cells that spans a microzone from side to side.

Input in a pattern not meeting this standard is not recognised. A failure to meet threshold does not trigger a graded response but no response at all, regardless of how close a match the input is in other regions, or which parts match and which do not. It is also immaterial how good the match is overall. There is no proportionate (or any other) response to a partial match. This is a key output and perhaps the central accomplishment of this design, because the response to a partial match would be arbitrary.

This is not in any way to suggest that Purkinje cells must be silenced to modulate firing of nuclear cells*.* The proposal is not that modulation is contingent on all-or-nothing firing, but all-or-none Purkinje cell coordination. It is unnecessary for a coordinated change in the rate to be a full pause. And while, in theory, uncoordinated firing could be in any permutation of rates, in practice firing of Purkinje cells omitted from a learned ‘pause’ is maintained at robust intrinsic rates [23, 49]—or higher, elevated by excitatory input. Poor coordination is not dysfunctional; simply, it exercises a default veto. A veto is the appropriate response to less-than-fully known input to a microzone.

So, patterns are learned, stored and confined to have an effect, at the indivisible level of a functional population of Purkinje cells, the microzone.

## What effect does a fully known pattern have?

Section 5 described the response where input to (any part of) a microzone is in an unknown pattern of active cells. This section describes the effect when a microzone receives a good match.

### Direct effect

It has been for a long time a central proposal of learning models that the function of training is to calibrate synaptic weights to make the learned response of Purkinje cells pattern dependent. It will be argued here on the contrary that the function is to *remove* a graded effect on Purkinje cell firing of synaptic modification, and that pattern recognition has a gating function.

A parallel fibre makes contact on one in every two Purkinje cells it passes at an average of 1.24 synapses, that is, 1 and sometimes 2 [22, 39], so a Purkinje cell receives input from a random sample of around half of a pattern of active cells (more accurately: from half the cells active within a learning-defined time window). The fixed density (and large number) of active cells means each pattern trains a predictable fraction of synapses on a Purkinje cell.

The density of parallel fibre activity, expressed as the fraction that are active, is the probability that a given synapse is active. This is therefore the probability, *p*, that a synapse participates in a particular pattern. The changing relative proportions of a stored pattern which overlap with 1 other pattern, with 2, with 3 and so on as more patterns are stored, is given by

$$ y=\frac{n!}{k!\left(n-k\right)!}\times {p}^k\times {\left(1-p\right)}^{n-k} $$

where *y* is the probability that a given synapse participates in *k* out of *n* stored patterns, and *p* is the fraction of parallel fibres that are active. *y* is also therefore the proportion of each pattern (the same for all of them) that overlaps with *k* other patterns, for *n* + 1 patterns stored, by the law of large numbers. In reality, *p* is a constant, assuming the density of parallel fibre activity is uniform and constant.

**Fig. 5:**
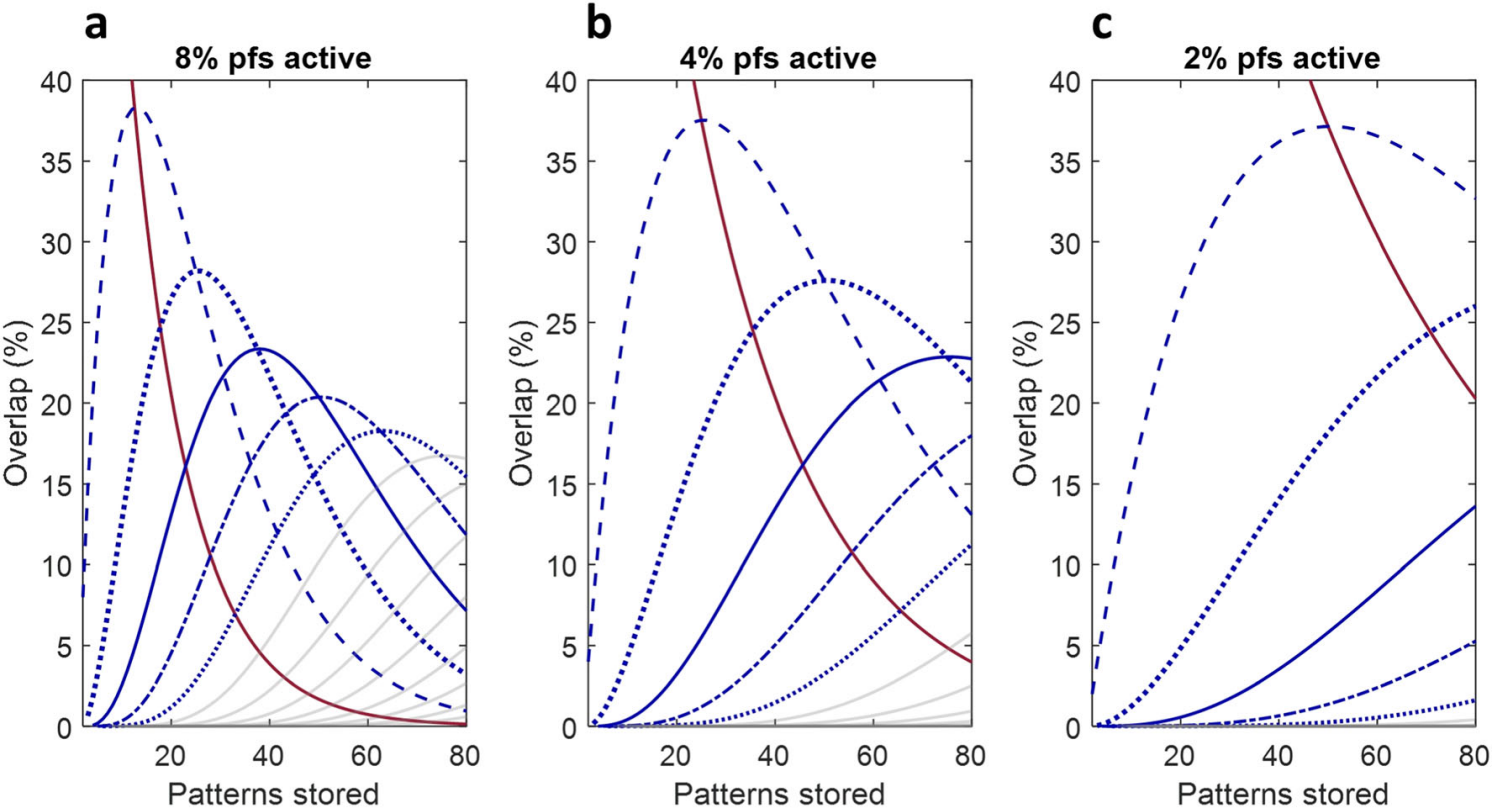
**a**–**c** The changing relative proportions of a stored pattern of parallel fibre input to a Purkinje cell which overlap with 1 other pattern, with 2, with 3 and so on as more patterns are stored, up to 13 in panel **a**, 8 in panel **b** and 5 in panel **c**. Eight percent of parallel fibres are active in panel **a**, 4% in panel **b** and 2% in panel **c**. The solid red line (all panels) is the proportion of a learned set of modified synapses that does not participate in other patterns. This is also (very near) the proportion of the total that participate in no patterns. Dashed line: the proportion which also participate in one other pattern; thick dotted line: 2 other patterns; solid line: 3; dots and dashes: 4; thin dotted line: 5. The solid faint grey lines in panels **a** and **b** show the proportion that participate in 6 other patterns, in 7, in 8 and so on, from left to right. The *x* range minimum is 2

Stored patterns therefore overlap in predictable numbers, simulated in Fig. 5. Each pattern contains the same number of synapses that also participate in 1 other stored pattern, and in 2, and 3 and so on. The numbers are a function of the number of patterns stored, and the proportions are the same for all stored patterns. The ratio of trained to naïve synapses is the same for all Purkinje cells trained to the same number of patterns and therefore all Purkinje cells in the same microzone (because climbing fibre lessons are received in a synchronised volley), and all microzones trained to the same number of patterns (which they may be if they have the same capacity and they are all trained to capacity).

An estimated 80–85% of parallel fibre-Purkinje cell synapses are strongly depressed, to the extent that there is ‘no detectable somatic response’ to granule cell stimulation ([27] p. 9676). This is consistent with a high estimate of ‘electrically silent’ synapses made by parallel fibres activated by cutaneous stimulation [16]. The Isope and Barbour detection threshold does not rule out compound responses (Boris Barbour, private correspondence dated 7 December 2018). But a collective effect, if it exists, would presumably be weak. Also, it would not be pattern-specific but generic, for the same reason that overlap is statistically predictable: most synapses also participate in a random sample of other patterns, subject to constraint of sample size by a probability distribution (Fig. 5a–c).

In our model, the function of learning is to block or uniformly (and greatly) weaken transmission. This does not mean synapses are all stripped of intrasynaptic AMPA receptors (although some are: [35]). Equality is not necessary between synapses but between (known) patterns. Simply, direct excitatory input in a known pattern is without a significant effect either because it is insignificantly weak or because it is made standard (and weak) at the scale of activity sampled by a Purkinje cell.

As a result, the response is independent of the particular pattern of input and of the distribution among active cells of the rates they each fire at. The whole class of known input patterns evokes the same functionally uniform response. The function of learning is to polarise synaptic weights. This is in order to prevent an effect of weights from interfering with control of output coding, and as such is directly contrary to the idea that learning adjusts weights to code output. Pattern recognition (in this contention) discriminates only between the class of known patterns and the residual (and unlimited) class of all other input.

### Indirect effect

Purkinje cells are interleaved with stellate cells, inhibitory interneurons that receive excitatory input from parallel fibres and inhibit Purkinje cells. Parallel fibre input to stellate cells drives feed-forward inhibition of Purkinje cells. The parallel fibre-stellate cell synapse learns in the same conditions as the parallel fibre-Purkinje cell synapse but the sign is reversed. That is, the parallel fibre-stellate cell synapse is potentiated by a course of training with paired input, while unpaired (parallel fibre only) input leads to synaptic depression (in vivo: [17, 31, 32], in vitro: [50, 56, 57]). Depression is severe, entirely blocking a postsynaptic effect (even with input at several 100 Hz, Henrik Jӧrntell, private correspondence dated 31 March 2017).

We propose that the effect of synaptic modification trained by experience in natural conditions is to polarise parallel fibre synaptic weights, as it is at the parallel fibre-Purkinje cell synapse. There is no direct evidence that transmission at climbing fibre-trained synapses is concentrated in a compressed range, as there is at the parallel fibre-Purkinje cell synapse. However, the number and timing of lessons is the same for both populations (in the same microzone). Polarisation would not mean transmission is the same strength at all synapses that participate in a learned pattern, but that there is no selective (i.e. variable) effect of synaptic weights on the response, at the collective level of parallel fibre activity that drives feed-forward inhibition. There is evidence that this is true.

Interneurons reflect ‘granule cell input with linear changes in firing rate’ ([29] p. 6), so that inhibition of Purkinje cells is at a rate controlled by parallel fibres. Moreover, this translates into a proportionate effect on Purkinje cells. The Purkinje cell firing rate has a linear relationship with the balance of excitatory input from parallel fibres and inhibitory input from interneurons. In mice, the ‘locomotion-dependent modulation of the balance between excitation and inhibition [of Purkinje cell dendrites] generates depolarising or hyperpolarising dendritic *V*_m_ [dendritic membrane voltage] changes that linearly transform into bidirectional modulation of PC SSp [Purkinje cell simple spike] output’ ([29] p. 9). Granule cell signals can be highly variable. Jelitai and colleagues measure the net effect of all inputs to a Purkinje cell, so that the results do not speak to the synapse-by-synapse effect of learning on transmission. However, at net level, the simple spike rate varies reliably and linearly with afferent rates and this relationship is sufficient to explain the data (so that there is no need for a hypothetical mechanism that uses an algorithm to control synaptic weights).

In the conditioned response, a known pattern is received exclusively at depressed synapses. With a direct excitatory effect absent (or the weakened/normalised equivalent), the balance shifts strongly to control of dendritic membrane potential by interneurons, themselves controlled by a linear relationship with granule cell rates.

## Instruction signals

### Climbing fibre discharge

Functionally, binary transmission would make hypothetically graded climbing fibre teaching signals unnecessary, because they are not needed to teach graded weights. Climbing fibres discharge in short bursts of spikes, originally reported to have an all-or-nothing signature [15]. The number of spikes is variable but in a small range and unpredictable on any particular occasion [12]. There has been a more recent move to argue—and is now probably the mainstream view [19, 51, 64]—that climbing fibre signals teach a graded lesson that depends on the number of spikes in a burst. We argue here for a rehabilitation of the all-or-nothing view, and that the evidence is robust.

Discharge within each burst is at an invariant high frequency [34, 36]. Over many bursts, the number of spikes correlates statistically with the timing of discharge relative to the phase of inferior olive subthreshold oscillation [36], leading to the idea that the number of spikes may code the oscillation phase, teaching graded parallel fibre synaptic modulation. The number was later reported to correlate with the amplitude, but not the phase, of oscillations [4]. The discrepancy is unresolved.

In fact, neither predicts the number of spikes in any given instance. Caution has been urged, even by supporters of a graded signal. The ‘number of spikes per CF [climbing fibre] burst was quite variable from one burst to the next and … the changes in burst size for any given situation were small (<1 spike per burst) and could only be detected in the average as a slight probability bias toward generating more bursts with many (>4) or few (1) spikes’ ([38] p. 4). Nor has a correlation been shown to be functional. If oscillations code anything, it is unknown what it is.

The number of spikes in a burst is very modest. The average number is 2–3 and the range is 1–6 [4, 36]. The number is further reduced because at least 3 spikes are needed to induce LTD [53], and further still if the number is inversely correlated to the amplitude of oscillations [4], which is larger among functionally grouped, strongly gap-junction-connected cells.

Moreover, olivary spikes do not propagate reliably. Spikes are generated in the initial axonal segment but some fail to propagate far (> 125 μm), so that what is initially a group of 4 (say) can become a group of 3, or two. Transmission failure is at random: the first spike always propagates, and the others propagate with variable probability (range *p* = 0.66–0.89; [36]), depending on their position in a burst. The probability that a burst contains a transmission failure also depends on the original size of the group—for example, an average of 2 out of 3 five-spike bursts are redacted (Fig. 2). The unpredictable failure of spikes to propagate means that if information is coded in the number of spikes initially generated, it is unreliably transmitted.

Finally, burst size does not code signals received as input to the inferior olive—‘The mean number of spikes … [was] independent of the stimulus intensity’ ([12] p. 201). This was found whether depolarisation is just over threshold or stronger, and has never been refuted. So, input data coded as excitatory rates are not represented in the number of spikes, even before spikes are lost to transmission failure. Nor is a graded lesson coded in the intraburst rate; interspike intervals are highly reliable, such ‘that the timing of spikes within a burst in the olivary axon is highly stereotyped…, with only the number of spikes varying’ ([36] p. 392).

So, graded climbing fibre instruction would need to be coded in a very modest number of spikes. Moreover, the number of spikes does not code signals that drive firing. Even if it did, spikes are not reliably transmitted—targets would receive the wrong lesson more often than not. The interspike interval is always the same, so information is not coded in the spike rate within a burst. Contrary to the graded view, all of this suggests more naturally that the climbing fibre signature is narrowly *constrained* in form. We propose instead that the all-or-nothing view is the correct interpretation.

### Climbing fibre LTD

The climbing fibre-Purkinje cell synapses is plastic *in vitro* [11, 20, 63]. Does that interfere with binary lessons?

Like parallel fibre-Purkinje cell LTD, climbing fibre LTD requires postsynaptic Ca^2+^ elevation and activation of group 1 mGluRs [20]. Climbing fibre LTD is reported to cause a reduction of climbing fibre–associated Purkinje cell dendritic calcium transients [63] which is sufficient to reverse the direction of plasticity at the parallel fibre-Purkinje cell synapse [11].

It is not known if climbing fibre LTD is functional *in vivo* or, if so, what function it has. Induction was by 5-Hz tetanisation for 30 s. This is in the upper range of olivary firing frequency*.* Sustained high frequencies cause poisonous levels of calcium. ‘High frequency Ca signals can reach neurotoxic levels when the frequency of complex spikes is transiently or persistently high’ ([55] p. 380). Possibly, transmission strength is self-depressing at high rates in order to forestall a toxic effect. If plasticity is bidirectional, as reported at climbing fibre synapses during postnatal development [7, 40], it may be that synapses depress until rates subside and then revert to normal.

Hence, climbing fibre LTD, if present in natural conditions, may have no sustained or function-impacting effect on parallel fibre synaptic strength. Indeed, a persistent effect might be expected to impair and not improve function. The reasons are as follows: (1) a function has not been reported (or proposed as part of a model of circuit function); (2) the high climbing fibre rate needed to induce it may be toxic; (3) it would subsequently alter *all* parallel fibre synaptic modification under climbing fibre tuition, indiscriminately; (4) such an impact would occur in later conditions to which the high climbing fibre rate did not relate; (5) assuming LTD is reversible, an effect on parallel fibre plasticity would be time-limited without a functional justification.

## Discussion

We propose that functionally grouped Purkinje cells learn to behave with coordinated timing, so that output of a circuit either is driven by coordinated behaviour of the whole population of a microzone, or else is blocked. Many brain regions face the challenge of simultaneously processing heavy throughput. In the cerebellum, the organisation and dimensions of a microzone allow circuits to present a large target to parallel fibres, and effectively orchestrates a functionally indivisible response. It is indivisible because there must be coordination of firing of the cells that control output, or there is no output at all.

We propose that pattern memory and control of output rates are separate functions, coded by different collective attributes of parallel fibre activity that vary independently. This allows the separate functions they serve to be carried out in parallel without either affecting the other, although they are intimately related (and, in active circuits, in constant operation).

Pattern recognition is a response to the binary (on/off) pattern of active parallel fibres. Each pattern, at any time—received across an entire population of Purkinje cells—is an effectively unique representation of mossy fibre input upstream, but all are otherwise equal in being randomly decorrelated and equally dense, so that an effect on the postsynaptic cell of the pattern *per se* is invariant. Given these properties, the make-up of a pattern—which cells are active and in what permutation—is immaterial, i.e., without effect. An effect is confined to firing rates, but here again, as rates contained in a pattern are randomly distributed among active cells (and synaptic weights are not graded), the particular distribution does not change the effect on the postsynaptic cell, nor therefore alter the response. Put another way, Purkinje cells do not remember (meaning: their response does not discriminate between) learned patterns individually. They discriminate only between learned patterns as a class, and the residual class of all other (therefore unknown) activity.

The second function of coding is control of Purkinje cell firing. The role of learning is partly to eliminate synaptic weights as a variable, by teaching transmission that is either very weak or robust. It is also to switch the balance of input to a Purkinje cell from control by excitation to control by inhibition, with learned timing provided by pattern memory. The theoretical attempt to explain a learning-modulated role of interneurons is nothing new [1, 18, 33]. In the present proposal, however, inhibition is not a response to a memory of patterns individually, and synaptic weights are not used to make graded adjustments to the balance of excitation and inhibition. Learning controls instead selection of circuits to have output, and timing, by gating the response.

For some years, there has been a strong influence on cerebellar theory of a large class of models (together the supervised learning model) which share the idea that the cerebellum implements a supervised learning algorithm (though they do not agree which one), and that individual Purkinje cells learn patterns that are stored as parallel fibre synaptic weights, the result of long-term synaptic modifications which accrue across training under climbing fibre tuition [1, 8, 14, 18]. Following training, learning supplants the naive response with a weighted response to input in a recognised pattern.

In this model, learning homes in on a ‘desired’ output, the learned response (hence ‘supervised’, to teach the correct response). The Purkinje cell firing rate (or in some models a spike) is a learned response to a remembered cue. The response depends not only on receiving input in a known pattern of active cells but on the rates at which they each fire, because each signal must receive bespoke modulation to collectively generate the correct output. In our model, the restriction to a desired output is absent, because pattern memory is uncoupled from control of Purkinje cell firing. All learned patterns can in theory evoke any rate of Purkinje cell firing, which can be different every time. This has the flexible advantage that a pattern is serviceable across a range of input and output rates. This would permit motor output, for example, at a pattern-selected phase of a movement cycle to respond proportionately to variation of input rates, so that is responsive to the rate of execution of movement, and step-by-step variability in cycle duration.

### Author Contribution

MG conceived the ideas, developed the models and wrote the paper. RCM helped develop the ideas, commented on previous drafts and edited the manuscript.

## Footnotes

1 ‘More observations would be needed to be sure’ that deep nuclei contain neurons that are ‘purely interneuronal, that is, without any axon leaving the CN [cerebellar nuclei]’ (Marylka Uusisaari, personal correspondence dated December 21 2018).
